# Sonographic evaluation of adrenal size in neonates (23 to 41 weeks of gestation)

**DOI:** 10.1186/s12887-018-1056-4

**Published:** 2018-02-14

**Authors:** Shigeo Iijima

**Affiliations:** Department of Neonatology, Toho University Medical Center, Tokyo, Japan

**Keywords:** Adrenal gland, Size, Adrenal gland area, Ultrasonography, Neonate

## Abstract

**Background:**

Fetal adrenal gland size is known to have a positive correlation with both gestational age and estimated body weight. In contrast, some clinical observations suggest that maturation of the adrenal stress response occurs after 30 weeks of gestation. In this study, adrenal gland size at birth in extremely preterm to term neonates was investigated using ultrasonography to evaluate the adrenal developmental pattern and the impact of prematurity and perinatal factors.

**Methods:**

The area of the right adrenal gland was measured in the first 3 h of life in 350 neonates and corrected for birth weight (BW) to determine the corrected adrenal area index (cAI). The neonates were subdivided into three groups: group 1 (before 30 weeks of gestation), group 2 (30 to 36 weeks), and group 3 (after 37 weeks). Differences in the cAI among the 3 groups were compared to estimate the impact of perinatal factors.

**Results:**

The adrenal gland size was measurable in all neonates with gestational age ranging from 23 to 41 weeks. Right adrenal gland area was highly correlated with BW (*r* = 0.75, *p* < 0.01). cAI showed a significant negative correlation with gestational age in group 1 (*r* = − 0.67, *p* < 0.01), whereas it showed no correlation with gestational age in both groups 2 and 3. As for the impact of perinatal parameters on cAI, only gestational age in group 1 and only fetal distress in group 2 were correlated with cAI. In group 3, perinatal parameters such as fetal distress and low Apgar score were correlated with cAI.

**Conclusions:**

The present study demonstrated that the developmental pattern of fetal adrenal gland was different before and after 30 weeks of gestation, suggesting that the magnitude of adrenal stress response might mature after 30 weeks of gestation.

## Background

With the advent of ultrasonography (US), many authors have studied the sonographic appearance and measurement of normal fetal adrenal gland in utero [1–4]. These studies showed that fetal adrenal gland size is positively correlated with both gestational age and estimated body weight. In neonates, US has been shown to provide a useful method of evaluating the appearance of the adrenal glands to diagnose pathologies such as adrenal hemorrhage and hyperplasia [5, 6]. However, regarding postnatal adrenal size, some authors reported sonographic evaluation in the late 1980s, but the focus was solely on the change in size postnatally, based on marked involution of the fetal zone component during the early weeks and months of extrauterine life [7, 8].

During the last two decades, the advances in the field of US have enabled higher resolution display and precise quantitative measurement of the organ dimensions.

Recently, it has been recognized that some very preterm infants develop acute profound primary circulatory failure that responds to glucocorticoid therapy, and a relationship between hypotension and relative adrenal insufficiency in preterm infants has been discussed [9, 10]. Previous findings demonstrated that preterm infants born before 30 weeks of gestation have a higher risk for the development of, not only chronic lung disease, but also early adrenal insufficiency in comparison to infants born after 30 weeks of gestation [5, 11].

The objective of the present study was to demonstrate adrenal gland size in extremely preterm to term neonates in a specially designed prospective study, and to compare adrenal gland size between preterm and term infants to estimate the impact of prematurity and perinatal factors.

## Methods

The author prospectively assessed the relationship between neonatal adrenal gland size, as measured by 2-dimensional (2D) US, and perinatal factors. Written informed parental consent was obtained.

### Patients

Neonates admitted immediately after birth to the neonatal intensive care unit (NICU) at Toho University Medical Center between April 1, 2009, and March 31, 2011, were eligible for the study. Exclusion criteria were as follows: 1) outborn; 2) major congenital anomalies; 3) evident intrauterine infections (e.g. cytomegalovirus infection); and 4) parental refusal of participation.

### Sonographic evaluation of the adrenal gland

**Fig. 1:**
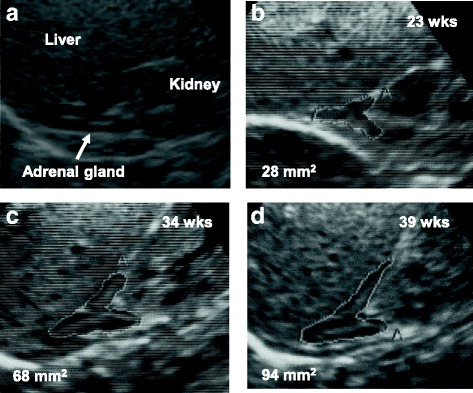
(**a**) Ultrasonographic scan of the right adrenal region of a representative neonate obtained immediately after birth by the longitudinal antero-lateral approach. Sonographic appearances of the right adrenal gland of a representative in each group: (**b**) group 1: a neonate born at 23 weeks of gestation, (**c**) group 2: a neonate born at 34 weeks of gestation, and (**d**) a neonate born at 39 weeks of gestation. The border of the adrenal gland was traced manually to calculate the adrenal gland area

All ultrasonographic scans were performed by an experienced operator (the author), using a Toshiba SSA-660A (Toshiba, Tokyo, Japan) ultrasound machine with a 7-MHz sector transducer within the first 3 h of life. With the infant in the supine position, longitudinal images of the adrenal glands were taken and the adrenal area was measured. The techniques for locating the adrenal glands and measuring the adrenal area were previously described (Fig. 1) [12]. Some researchers previously reported less variability in right than in left adrenal gland [8, 12, 13]. Therefore, only the right adrenal was used for all measurements to minimize technical errors that could influence the results.

### Sonographic data analyses

It was previously described that fetal adrenal gland size was positively correlated with estimated body weight in ultrasonographic studies [1–4]. Moreover, in the previous study, we demonstrated that the adrenal area at birth was positively correlated with birth weight (BW) [12]. Therefore, a corrected adrenal area index (cAI) was calculated as the ratio of adrenal area to BW (mm^2^/kg) to investigate the correlation with gestational age (GA) and perinatal parameters. Based on the previous finding that preterm infants born before 30 weeks of gestation have a higher risk of developing adrenal insufficiency compared with those born after 30 weeks of gestation [5, 11], infants were subdivided into three groups: preterm infants born before 30 weeks of gestation (group 1), preterm infants born between 30 and 36 weeks of gestation (group 2), and term infants born after 37 weeks of gestation (group 3). Differences in the cAI among the 3 groups were analyzed.

### Clinical parameters

Collected maternal data included complications during pregnancy and conditions surrounding delivery, such as premature rupture of membranes (PROM), preeclampsia, use of antenatal steroids, fetal distress, and mode of delivery (cesarean section or not). Collected infant data included GA, BW, sex, Apgar score, and small for gestational age (SGA). The GA at birth was determined by maternal history based on the last menstrual period and obstetric examination with ultrasonography. PROM and preeclampsia were diagnosed clinically by the attending obstetrician. Fetal distress was diagnosed by intrapartum fetal heart rate irregularities, meconium staining of amniotic fluid, and abnormal fetal movement. The Apgar score was assessed at 1 and 5 min, and low 1-min Apgar score was defined if the Apgar score at 1 min was 6 or lower. SGA was defined as both BW and birth length below the 10th percentile for GA based on standards of birth size for Japanese neonates [14].

### Statistical analysis

All statistical analyses were conducted using the Statistical Package for Social Sciences, version 18 (SPSS, Tokyo, Japan) for Windows. The Kolmogorov-Smirnov test was used for data normality testing. Data were presented as median (interquartile range) and percentage. The relation between continuous variables was investigated by the Pearson’s and Spearman’s rank correlation coefficients. Differences between both groups were compared using the Mann-Whitney U test, and Kruskal-Wallis one-way analysis of variance on ranks was used to compare the overall differences among the 3 groups. A *p* value < 0.05 was considered statistically significant.

## Results

A total of 374 neonates were admitted to our NICU during the study period between April 2009 and March 2011, and 350 of these neonates met the inclusion criteria. Among the excluded 24 infants, 17 were outborn, 5 had major congenital anomalies, and the parents of 2 did not agree to participate. Two infants had intrauterine cytomegalovirus infection with severe brain anomaly and were excluded because of the presence of major congenital anomalies. There was no infant whose adrenals were not ultrasonographically examined in the first 3 h of life. In the enrolled 350 consecutive neonates (male/female ratio, 172:178), the median GA was 36.3 weeks, with an interquartile range (IQR) of 33.5 to 38.4 weeks, and the median BW was 2156 g, with an IQR of 1744 to 2847 g. Among the subjects, none was subsequently diagnosed with congenital adrenal hyperplasia, adrenal hemorrhage, or primary adrenal tumor (e.g. neuroblastoma).

### Sonographic appearance

The adrenal gland was identified in a suprarenal location in all neonates (100%), without difficulties. However, the right adrenal was consistently easier to identify in its entirety than was the left. The glands were visualized in the configuration of a “V” or “Y” with a characteristic echogenicity of a thin, central hyperechoic stripe surrounded by a thicker hypoechoic rim. The appearance of the adrenal gland was not different by gestational age (Fig. 1). The central hyperechogenic area was not identified only in the cases whose gestational age was 23 weeks.

### Measurement of right adrenal gland area

In all subjects, the median adrenal gland area was 71.0 mm^2^, with IQR of 35.3 to 94.0 mm^2^. As for the correlations between the adrenal area and BW, a strong correlation was observed between BW and adrenal area (*r* = 0.75, *p* < 0.001).

**Fig. 2:**
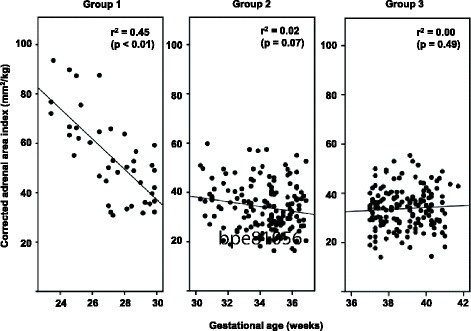
Scatter plots showing the correlations between the corrected adrenal gland area index and gestational age in each group. Group 1: preterm infants born before 30 weeks of gestation, group 2: preterm infants born between 30 and 36 weeks of gestation, group 3: term infants born after 37 weeks of gestation

The data on cAI were compared among the 3 groups. cAI was significantly higher in group 1 than in groups 2 and 3, and there was no difference between groups 2 and 3 (Table 1). Next, the cAI was plotted against GA in each group (Fig. 2). As a result, two different patterns of the relationship were observed between group 1 and the other groups. The cAI was negatively correlated with gestational age in group 1 (*r* = − 0.67, *p* < 0.001), whereas it was not correlated to gestational age in groups 2 (*r* = − 0.14, *p* = 0.07) or 3 (*r* = 0.05, *p* = 0.49).

**Table 1:** Clinical characteristics and adrenal size of the study population (*n* = 350)

	Group 1 (*n* = 41) Gestational age < 30 wk	Group 2 (*n* = 154) Gestational age 30 - 36 wk	Group 3 (*n* = 155) Gestational age > 36 wk	*P* value
Neonatal characteristics				
Gestational age, wk	27.3 (25.2–29.1)	34.6 (33.2–35.7)	39.0 (37.7–39.9)	< 0.01
Birth weight, g	849 (622–1206)	1970 (1670–2203)	2886 (2264–3260)	< 0.01
Sex, male: female	10: 31	85: 69	77: 78	< 0.01
1-min Apgar score	6 (3–7)	8 (7–9)	8 (8–9)	< 0.01
5-min Apgar score	7 (6–8.5)	9 (9–9)	9 (8–9)	< 0.01
Low 1-min Apgar score, *n* (%)	29 (71)	23 (15)	28 (18)	< 0.01
Small for gestational age, *n* (%)	13 (32)	34 (22)	36 (23)	0.34
Maternal characteristics				
Maternal preeclampsia, *n* (%)	12 (29)	23 (15)	5 (0.3)	< 0.01
Premature rupture of the membrane, *n* (%)	16 (39)	56 (36)	22 (14)	< 0.01
Antenatal steroids, *n* (%)	31 (76)	67 (44)	0 (0)	< 0.01
Tocolysis, *n* (%)	26 (63)	99 (64)	0 (0)	< 0.01
Fetal distress, *n* (%)	10 (24)	22 (14)	34 (22)	0.12
Cesarean section, *n* (%)	29 (71)	98 (64)	52 (34)	< 0.01
Adrenal size of the neonates				
Adrenal area at birth, mm^2^	47.0 (37.0–53.5)	64.0 (51.0–76.3)	93.0 (75.0–112.0)	< 0.01
Corrected adrenal area index at birth, mm^2^/kg	51.1 (38.1–65.2)	33.3 (26.8–40.0)	33.9 (28.4–38.7)	< 0.01

Values are presented as median (interquartile range) or number (percentage). Low 1-min Apgar score was defined if the Apgar score at 1 min was 6 or lower

### Influence of perinatal parameters on adrenal area index

**Fig. 3:**
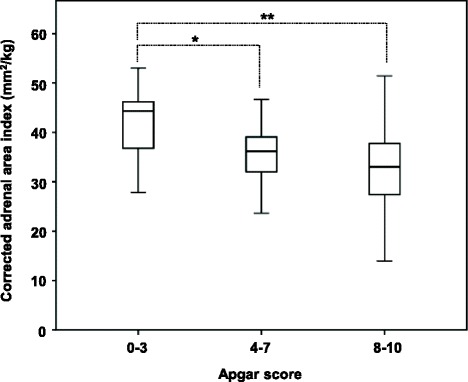
Box and whisker plot showing the results of corrected adrenal gland area index for the 155 term infants with 1-min Apgar score of 0-3, 4-7, and 8-10. Each box ranges from the 25th percentile at the lower edge to the 75th percentile at the upper edge. The median is shown as a line across the box. The two adjacent values below and above the box are: the largest value is below the upper inner limit and the smallest value is above the lower inner limit

Clinical and sonographic data of the 3 groups are summarized in Table 1. As for the comparison of adrenal sonographic data among the 3 groups, differences were significant for all perinatal parameters except for SGA and fetal distress. However, for the intragroup correlation study, only gestational age in group 1 and only fetal distress in group 2 were correlated with cAI. In group 3, perinatal parameters such as fetal distress, Apgar score, and low 1-min Apgar score were associated with cAI (Table 2). In this subgroup, cAI was greater in neonates with 1-min Apgar score 0-3 (median: 44.3 mm^2^/kg) than in those with 1-min Apgar score 7-10 (median: 33.0 mm^2^/kg) (*p* < 0.01); cAI was also greater in neonates with 1-min Apgar score 0-3 than in those with 1-min Apgar score 4-6 (median: 36.2 mm^2^/kg) (*p* = 0.04) (Fig. 3).

**Table 2:** Correlation between corrected adrenal area index and perinatal parameters in each group

	Group 1		Group 2		Group 3	
	CC	*P* value	CC	*P* value	CC	*P* value
Neonatal characteristics						
Gestational age	−0.67	< 0.01	−0.15	0.07	0.06	0.49
Male sex	−0.31	0.05	0.08	0.33	0.06	0.49
1-min Apgar score	0.30	0.06	−0.06	0.47	−0.24	< 0.01
5-min Apgar score	−0.31	0.05	−0.11	0.18	−0.12	0.15
Low 1-min Apgar score	0.35	0.02	0.07	0.38	0.24	< 0.01
Small for gestational age	0.07	0.67	0.01	0.96	−0.08	0.31
Matemal characteristics						
Maternal Preeclamsia	−0.16	0.33	−0.02	0.81	−0.05	0.56
Premature rupture of the membrane	0.12	0.45	0.13	0.10	0.10	0.21
Antenatal steroids	0.06	0.73	0.14	0.10	–	–
Tocolysis	0.16	0.29	0.07	0.39	–	–
Fetal distress	−0.02	0.89	0.21	0.01	0.21	< 0.01
Cesarean section	−0.15	0.34	0.03	0.67	0.04	0.64

Group 1: Gestational age < 30 weeks (*n* = 41); Group 2: gestational age 30-36 weeks (*n* = 154); Group 3: gestational age > 36 weeks (*n* = 155). Low 1-min Apgar score was defined if the Apgar score at 1 min was 6 or lower. *CC,* Correlation coeficient

## Discussion

The measurement of neonatal adrenal gland has been the subject of only a few previous ultrasonographic studies, with the latest report published in the early 1990s [7, 8, 13]. Various methods of measuring the adrenal size using US have been described because of the irregular shape of the gland. These include measurements of adrenal length, width, and circumference [7, 8, 13, 15–17]. In the present study, the adrenal area measurements were chosen. The author found only one study about the evaluation of adrenal size by adrenal area, which was conducted in the 1980s [8]. In addition to the multipart shape of the gland, insufficient resolution of the US device at that time resulted in difficulty in obtaining accurate information on the anatomical size of the glands. With the advent of the resolution and trace technology of US, it was possible to measure the adrenal area with higher accuracy than in the past. Therefore, as a measurement parameter to estimate the actual adrenal size, adrenal area might be preferable to adrenal length or width. Previously, we evaluated adrenal size by measurements of the adrenal area and demonstrated that the adrenal area could be measured in all neonates born after 23 weeks of gestation [12]. In the present study, although the author evaluated a larger number of subjects than in the previous study, the right adrenal gland was seen and measured in the assessment, without difficulty, in all study subjects.

In the present study, cAI was calculated to investigate the correlation with GA. A peculiar pattern was found between cAI in preterm infants with a GA below 30 weeks and in those with a GA above 30 weeks. cAI was negatively correlated with GA up to 30 weeks, whereas it remained almost constant from 30 weeks to term. Previously, Kangaloo et al. demonstrated in their ultrasonographic study that the length of the adrenal gland was directly correlated with GA up to 32 weeks of gestation, after which no significant increase in the length of the gland occurred to full term [13]. There might be some functional differences in fetal adrenal gland around 30 weeks of gestation. In the present study, however, the intergroup and intragroup comparisons among the 3 groups could not identify any significant perinatal factors that could explain why the correlation patterns between cAI and GA differed between before and after 30 weeks of gestation.

The fetal adrenal gland develops and exhibits a marked change in size, structure, and function during the fetal and perinatal periods. The fetal adrenal cortex consists of three compartments: the relatively large, inner fetal zone (FZ), the outer definitive zone (DZ), and the intermediate transitional zone (TZ). The FZ mainly produces androgens, the DZ produces mineralocorticoids, and the TZ contains enzymes for cortisol production [18]. The fetal adrenal cortex grows rapidly from approximately 10 weeks of gestation to term almost entirely owing to the enlargement of the FZ. Subsequently, the adrenal size almost equals that of fetal kidneys by 20 weeks of gestation, and the weight doubles between 20 and 30 weeks [19]. In contrast, the DZ growth occurs owing to hyperplasia by 10 to 12 weeks of gestation. Subsequently, the TZ cells, which differentiate from the definitive cortex, have the capacity to synthesize cortisol. By 30 weeks of gestation, the DZ and TZ begin maturation into the zona glomerulosa and the zona fasciculata, respectively [19]. Fetal adrenal size exhibits disproportionality relative to body weight throughout the entire growth process. The present study plainly showed that fetal adrenal growth might greatly change after 30 weeks of gestation.

Considering adrenal functions, Bolt et al. showed in their study that the cortisol response to adrenocorticotropic hormone stimulation in infants born before 30 weeks of gestation was significantly lower than in more mature infants [20]. Moreover, Heckmann et al. demonstrated that the severity of illness did not have a significant influence on cortisol production rates in preterm infants born before 30 weeks of gestation, and they concluded that cortisol production rates in such immature infants might reflect inadequate stress reaction [21]. Grofer et al. demonstrated a maturation of the adrenal stress response in infants born after 30 weeks of gestation by showing a positive association between severity of illness and cortisol production rate in those infants, unlike in more immature infants [22]. The hormonal demands of growth and maturation in premature infants need to be balanced with those of acute critical stress. However, this process is not yet complete in a preterm infant born before 30 weeks of gestation. Fetal mechanisms regulating glucocorticoid homeostasis might persist after birth to protect such immature infant from excessive glucocorticoids [23]. Recently, relative adrenal insufficiency has been discussed in the preterm infant born before 30 weeks of gestation [24]. This could be explained by the above-mentioned immature adrenal function of such preterm infants. It is thought that the developmental process of fetal adrenal function might support the change of the cAI shown in the present study. A limitation of this study is that the data collected about the mode of delivery included only whether cesarean section (CS) was performed or not, rather than including all the details. Especially in extremely preterm delivery, CS is often performed after the onset of labor. Turan et al. have suggested that the premature onset of labor has a relationship with fetal adrenal gland enlargement [4]. Therefore, the result may be influenced by the fact that the delivery occurs before or after the onset of labor.

In this study, especially in term neonates, there were significant correlations between cAI and both fetal distress and low 1-min Apgar score, that is, adrenal enlargement was associated with a clinical history of perinatal distress. A similar sonographic feature has been previously reported by some investigators [25–27]. Their histological studies postulated that adrenal enlargement was caused by adrenal congestion. Perinatal stress like asphyxia causes a redistribution of cardiac output to preserve blood flow to the more vital organs such as the brain, heart, and adrenal glands, and the consequent congestion may increase the pressure within the capillary network [27]. As hypoxic events cause damage to endothelial cells and impair vascular autoregulation, hemorrhage or infarction can occur in these organs, and probably in the adrenal gland [27]. However, the pathophysiology is not fully understood. Further investigations to demonstrate the causal relationship between perinatal asphyxia and adrenal size are warranted.

## Conclusions

The present study demonstrated that the developmental pattern of fetal adrenal gland was different before and after 30 weeks of gestation. In addition, the adrenal area relative to body weight correlated with perinatal asphyxia after 30 weeks of gestation. The results in the present study suggest that the magnitude of adrenal stress response might mature after 30 weeks of gestation.

## Abbreviations

2D: 2-dimensional
BW: Birth weight
cAI: Corrected adrenal area index
DZ: Definitive zone
FZ: Fetal zone
GA: Gestational age
IQR: Interquartile range
NICU: Neonatal intensive care unit
PROM: Premature rupture of membranes
SGA: Small for gestational age
TZ: Transitional zone
US: Ultrasonography
